# Unique Presentation of Rosai-Dorfman Disease as Concomitant Appendiceal and Rectal Masses with IgG4-Positive Plasma Cells Diagnosed by Core Needle Biopsy

**DOI:** 10.1155/2020/8814871

**Published:** 2020-10-09

**Authors:** Jenna J. Poldemann, Benjamin H. Hinrichs, Abouelmagd Makramalla

**Affiliations:** University of Cincinnati Medical Center, USA

## Abstract

Rosai-Dorfman disease (RDD), or sinus histiocytosis with massive lymphadenopathy, is a rare non-Langerhans cell histiocytosis. We report a case of a 69-year-old male with concurrent appendiceal and rectal masses who underwent CT-guided percutaneous biopsy. Histopathology confirmed a diagnosis of RDD with IgG4-positive plasma cells. It is believed to be a subset of RDD that shares similar features with IgG4-related disease suggesting some overlap of the two diseases. Because gastrointestinal RDD accounts for less than 1% of extranodal disease, it is important to recognize this entity in order to guide management. We review the presentation, diagnosis, and treatment of gastrointestinal RDD and discuss the possible overlap with IgG4-related disease.

## 1. Introduction

Rosai-Dorfman disease (RDD), also known as sinus histiocytosis with massive lymphadenopathy, is a rare non-Langerhans cell histiocytosis, first observed in 1965 by Destombes and established as a clinicopathological disease by Rosai and Dorfman in 1969 [1, 2]. The prevalence is reported as 1 : 200,000 with 100 new cases in the United States annually [3]. RDD classically presents as massive, painless bilateral cervical lymphadenopathy, low-grade fever, and weight loss, mainly in children or young adults. However, approximately 43% of cases present with extranodal disease without associated lymphadenopathy [3]. Gastrointestinal tract involvement accounts for less than 1% of extranodal cases. Nonspecific fibroinflammatory lesions are commonly seen in extranodal RDD with stromal sclerosis and emperipolesis. Characteristic histocytes are S-100+, CD68+, and CD1a- and demonstrate variable frequency of emperipolesis. It has recently been discovered that RDD has similar features as immunoglobulin (Ig) G4-related disease and there is speculation of some overlap between a subset of RDD and IgG4 disease.

## 2. Case Report

We report the case of a 69-year-old male with a past medical history of hypertension, coronary artery disease, ischemic cardiomyopathy, noninsulin dependent type 2 diabetes mellitus, and alcohol dependence who presented to our institution in cardiac arrest and underwent emergent cardiac catheterization. During admission, laboratory results were notable for anemia with a hemoglobin of 4.6 g/dL, compared to a baseline hemoglobin of 14.2 g/dL approximately four years prior, with mean corpuscular volume of 65 fL. Further workup revealed iron levels of less than 10 *μ*g/dL and ferritin of 3.3 ng/mL, confirming a diagnosis of iron deficiency anemia. CT of the chest, abdomen, and pelvis with intravenous contrast was performed to evaluate for occult malignancy.

**Figure 1:**
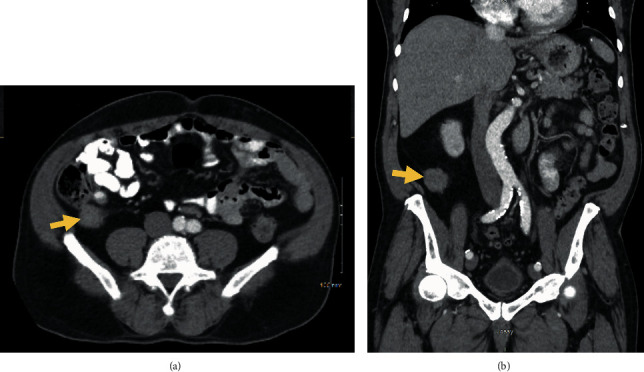
(a) Axial abdomen/pelvis CT showing a soft tissue mass (arrow) at the tip of the appendix. (b) Coronal image of the soft tissue mass (arrow) at the tip of the appendix.

**Figure 2:**
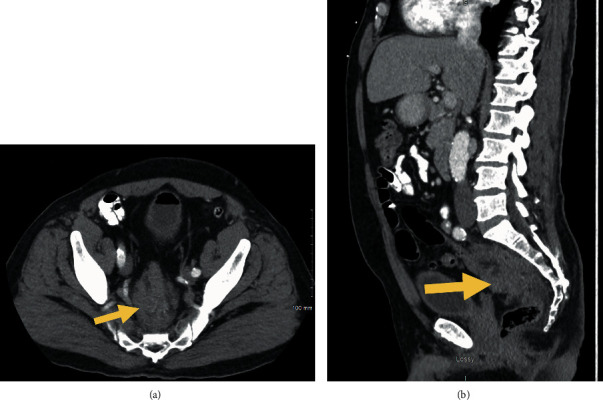
(a) Axial abdomen/pelvis CT showing a soft tissue mass (arrow) inseparable from the rectal wall. (b) Sagittal abdomen/pelvis CT showing a soft tissue mass (arrow) inseparable from the rectal wall.

**Figure 3:**
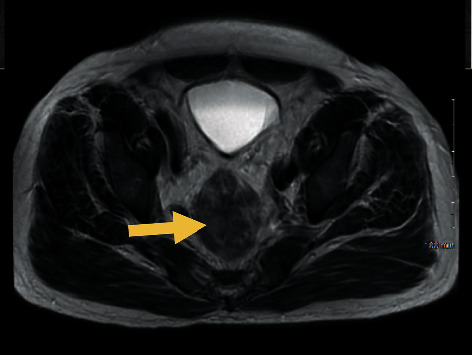
Axial image of MRI pelvis showing a nonspecific rectal mass (arrow).

While chest CT did not show any findings of intrathoracic malignancy, abdomen and pelvis CT showed a 2.4 × 3.2 cm soft tissue mass at the tip of the appendix (Figure 1) as well as a 5.3 × 2.5 cm lobular perirectal mass (Figure 2) which were concerning for a malignancy. There were also borderline enlarged perirectal and pelvic lymph nodes. MRI of the pelvis was also performed again demonstrating an infiltrative rectal soft tissue mass which was nonspecific (Figure 3).

A colonoscopy was performed with a normal appearing appendiceal orifice and normal rectal mucosa without any masses identified. The patient also underwent a flexible sigmoidoscopy with endoscopic ultrasound evaluation of the rectum. No rectal mass was seen on endoscopic ultrasound, and only small, benign appearing perirectal lymph nodes were identified and therefore, no biopsies were obtained.

**Figure 4:**
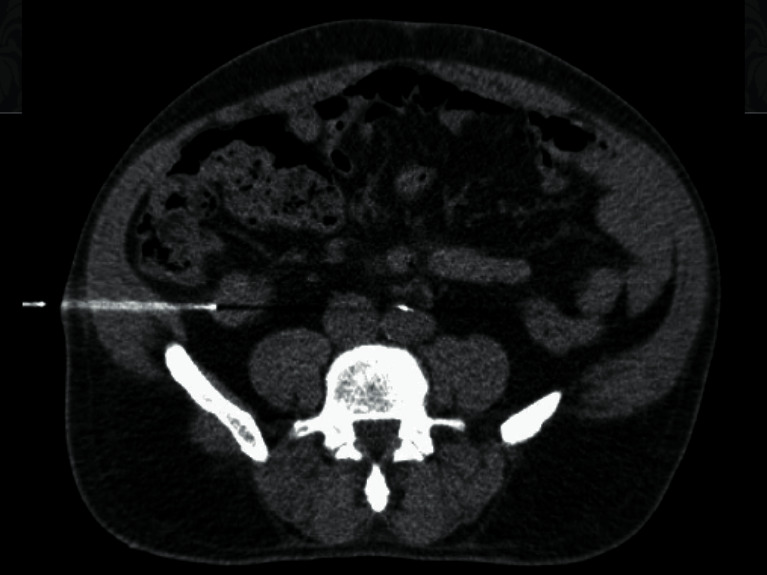
CT-guided core needle biopsy of the appendiceal mass.

Carcinoid tumor was in the differential in addition to malignancy. However, serologic studies showed normal gastrin (39 pg/mL), chromogranin A (2 nmol/L), and serotonin (86 ng/mL) levels. Given the presence of concurrent appendiceal and rectal masses, tissue sampling was further pursued for treatment planning. Three 18-gauge (Figure 4) core needle biopsies of the appendiceal mass were obtained and submitted to the pathology department for evaluation. We believe this is the first reported case of a CT-guided core needle biopsy of an extranodal gastrointestinal RDD.

**Figure 5:**
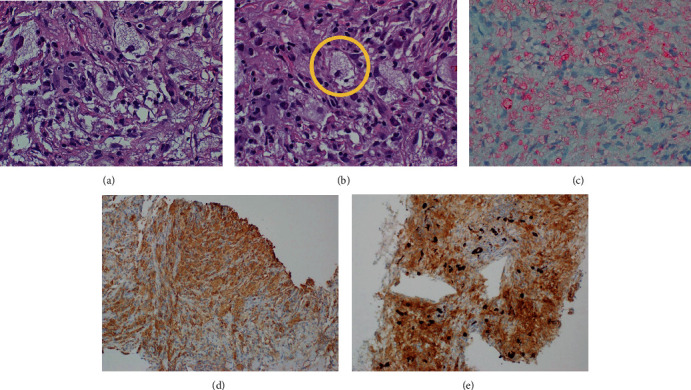
(a) Histiocytes with characteristic prominent nucleoli and pale abundant cytoplasm (hematoxylin and eosin, 600x). (b) Histiocytes with emperipolesis (hematoxylin and eosin, 600x). (c) Immunohistochemistry of the appendiceal mass with S-100 highlighting large histiocytes with prominent nucleoli (600x). (d) Immunohistochemistry of the appendiceal mass with CD68 highlighting large areas of histiocytes. (e) Immunohistochemistry of the appendiceal mass with IgG4-positive cells.

Histologic sections showed a heterogeneous lesion composed predominantly of histiocytes with numerous scattered plasma cells and lymphocytes (Figure 5(a)). Many of the histiocytes showed enlarged nuclei with prominent red nucleoli. Several foci of emperipolesis were also identified (Figure 5(b)). The accompanying plasma cells were predominantly scattered but focally aggregated. No atypical plasma cells were seen. A broad differential was considered and immunohistochemistry (IHC) performed to evaluate for RDD and IgG4-related sclerosing disease, as well as to rule out carcinoma, inflammatory myofibroblastic tumor, and Mycobacterium or fungal infection. The histiocytes with large nuclei and prominent nucleoli were positive for S-100 (Figure 5(c)) and CD68 IHC (Figure 5(d)) with CD68 also highlighting large areas of histiocytes. IgG4 IHC staining showed significantly increased positive cells (approximately 53 IgG4-positive cells) in a single 400x field (Figure 5(e)) in a background of predominantly scattered and focally aggregated positive cells. Overall, the diagnostic workup was most consistent with RDD, without evidence of carcinoma. Given the focally increased IgG4 staining, a component of IgG4-related sclerosing disease could not be entirely ruled out, with possible overlap between the two entities entertained by the pathologist.

The patient was discharged in stable condition and with an outpatient referral to an oncologist and a histiocytosis specialist at an outside institution. We were unable to obtain further workup or treatment.

## 3. Discussion

Rosai-Dorfman disease is an idiopathic proliferation of non-Langerhans cell histiocytes presumed to be a reactive inflammatory process [4]. Although the etiology is uncertain, some studies suggest a possible association with viral infections such as herpes viruses, Epstein-Barr virus, cytomegalovirus, and HIV but this has not been substantiated [5]. Recent studies also demonstrated gene mutations RDD patients which include NRAS, KRAS, MAP 2K1, and ARAF [3]. Studies have also reported associations with inherited diseases, neoplasms, and autoimmune diseases [3].

Gastrointestinal RDD causes subacute symptoms with gradual progression, typically in middle-aged females [3]. Patients may be asymptomatic with incidentally detected lesions or may present with abdominal pain, constipation, hematochezia, anemia, or bowel obstruction. The majority of reported cases of gastrointestinal RDD have been distal to the pylorus. The manifestations may be solitary or segmental and can present with or without associated lymphadenopathy.

Diagnosis of RDD can be made based on clinical history and histopathologic evaluation. Large histiocytes with abundant eosinophilic cytoplasm are characteristic of RDD with large hypochromatic nuclei and prominent nucleoli. The presence of emperipolesis (lymphocytophagocytosis) is helpful but not necessary for diagnosis. Emperipolesis can also be seen in Erdheim-Chester disease, Juvenile xanthogranuloma, or even malignant histiocytosis. On immunohistochemistry, histiocytes in RDD are positive for S100 and CD68, with variable positivity in CD163 and CD14 [3]. Histocytes are negative for CD1a and CD207, which distinguishes RDD from Langerhans cell histiocytosis. There is also a subset of extranodal RDD, usually involving the liver, lungs, or colon that has increased the number of IgG4-positive cells. In a study performed by Liu et al., approximately 30% of RDD cases had sclerosis and IgG4 plasmacytosis, typical of IgG4-related disease [5]. While there is no consensus of a cutoff for diagnosing IgG4-related disease, an IgG4/IgG ratio > 40% and greater than 10 IgG4+ cells/hpf has been suggested [6]. Approximately 30% of RDD cases studied by Zhang et al. had greater than 10 IgG4+ cells/hpf and >40% gG4/IgG ratio [7]. While a definite link has not been confirmed, evaluating the IgG4/IgG ratio in RDD patients is recommended as it is postulated that there is overlap between these two entities.

There is no systematic approach to determining treatment for RDD. 20-50% of cutaneous or nodal RDD cases are self-limited [7, 8]. Surgical excision can be performed for unifocal disease or cases of obstruction and is most effective in treating cutaneous RDD. Treatment with steroids, usually in higher doses than other autoimmune disease, has variable responses. Other treatments with possible efficacy include chemotherapy, sirolimus, immunomodulatory therapy, imatinib, or radiotherapy.

Our patient presented due to an acute coronary event with iron deficiency anemia and had incidentally detected multifocal gastrointestinal masses found on imaging, involving the appendix and rectum, with borderline enlarged pelvic and perirectal lymph nodes. A gastrointestinal malignancy with metastasis was initially the primary consideration. Despite distinctive soft tissues masses seen on CT and MRI, interestingly, the rectal mass was inconspicuous on endoscopic ultrasonography. This suggests that caution should be taken when using ultrasonography to evaluate disease. Additionally, it is likely that inflammatory changes related to RDD in the periappendiceal and perirectal soft tissues mimicked soft tissues masses on certain imaging modalities. Given the multifocal involvement, a percutaneous core biopsy of the lesion, rather than surgical excision, was performed for treatment planning. While only the appendiceal mass was biopsied, the perirectal soft tissue mass was felt to represent the same fibroinflammatory lesion based on imaging and clinical findings. Pathologic findings were typical for RDD with increased IgG4 plasma cells. Further studies are needed to evaluate significance of IgG4 plasmacytosis and delineate an algorithm for treatment.
